# High-Grade Non-Muscle Invasive Bladder Cancer: When to Move to Early Radical Cystectomy?

**DOI:** 10.7759/cureus.19399

**Published:** 2021-11-09

**Authors:** Raed A Azhar, Anmar M Nassir, Hesham Saada, Sameer Munshi, Musab M Alghamdi, Ahmad M Bugis, Mohamed A Elkoushy

**Affiliations:** 1 Department of Urology, King Abdulaziz University Faculty of Medicine, Jeddah, SAU; 2 Department of Surgery, Umm Al-Qura University, Makkah, SAU; 3 Department of Urology, King Abdullah Medical City, Makkah, SAU; 4 Department of Urology, International Medical Center, Jeddah, SAU; 5 Department of Urology, King Abdulaziz University Hospital, Jeddah, SAU; 6 Department of Urology, Suez Canal University, Ismailia, EGY

**Keywords:** survival, radical cystectomy, urothelial malignancy, transurethral resection, nonmuscle invasive, bladder cancer

## Abstract

Objectives

To compare the outcomes of bladder preservation therapy with early or deferred radical cystectomy (RC) in high-grade non-muscle invasive bladder cancer.

Methods

Prospectively collected data were obtained for patients undergoing transurethral resection of bladder tumor (TURBT) at a tertiary care center between 2007 and 2018. Patients with a high-grade tumor (HGT1) were divided into three groups, depending on the treatment plan: conservative (GI), early RC (GII), or deferred RC (GIII). Kaplan-Meier analysis was performed to assess the cancer-specific survival (CSS).

Results

Seventy-one patients were included, and the patients had a median (range) age of 49 (32-72) years. The GI, GII, and GIII groups included 34 (47.9%), 14 (19.7%), and 23 (32.4%) patients, respectively. A significantly lower number of GII patients underwent >2 TURBTs (14.3% vs. 100%, p<0.001). Compared to GIII patients, GII patients had a shorter time to RC from the initial diagnosis (5.7 vs. 36.2 months, p=0.03). Ileal conduit and orthotropic bladder diversions were comparable between both groups, with significantly higher postoperative complications in GIII patients. The median (IQR) follow-up times for the groups were 84 (49-102), 82 (52-112), and 73 (36-89) months, respectively. The five-year and 10-year CSS for GII and GIII patients was 79% vs. 75% and 78% vs. 64%, respectively (log rank=0.19).

Conclusion

Early RC should be considered an alternative treatment option in selected patients with HGT1 BC with expected longer life expectancy, which may significantly decrease postoperative complications and improve the CSS. However, selection bias in the current retrospective study may influence these outcomes.

## Introduction

Bladder cancer (BC) has high morbidity and mortality, with 549,393 new cases diagnosed worldwide in 2018 and 199,922 deaths [1-2]. Most newly diagnosed cases of BC are non-muscle invasive (NMIBC) [3-4]. However, carcinoma in situ (CIS) and high-grade T1 (HGT1) cancer is an aggressive disease with a 23-74% tendency for recurrence and 50% progression to muscle-invasive disease, even after patients have received intravesical therapy [5-7]. Of interest, clinical understaging and persistence of the disease are common at initial transurethral resection, which necessitates a second-look resection for patients with HGT1.

This aggressive behavior of HGT1 tumors significantly limits the effectiveness of bladder preservation strategies, including transurethral resection of the tumor (TURBT) followed by intravesical Bacillus Calmette-Guerin (BCG), which decreases the recurrence and progression rates [8]. Prior recurrence, number of tumors, and tumor size are important factors for recurrence while progression is influenced by the T-stage, presence of CIS, and grade [9]. Therefore, radical cystectomy (RC) was adopted as another alternative treatment option with a better chance of cure in these patients. Several studies have concluded that RC is the preferred treatment option due to better survival rates [10-11].

RC is considered an overtreatment in some patients and is associated with a high perioperative risk of morbidity and mortality; in addition, there is a substantial impact of the urinary diversion on the health-related quality of life. On the other hand, delayed RC might increase the risk of metastases and even bladder cancer-specific mortality, especially because one-third of patients treated with intravesical BCG will need RC during follow-up [12-13].

The American Urology Association (AUA) guidelines recommend an initial RC in patients with HGT1 tumors associated with CIS or lymphovascular invasion, in high-risk patients with persistent HGT1 on repeated resection, and in patients with a persistent or recurrent disease within one year following two induction courses, with or without maintenance treatment, of BCG. The European Association of Urology (EAU) and the National Comprehensive Cancer Network (NCCN) guidelines recommend an initial RC in patients with the highest risk of progression, including the patients with HGT1 with concurrent CIS and the patients with large multifocal or recurrent HGT1 with CIS in the posterior urethra. RC is also recommended for patients who fail BCG induction therapy either due to intolerance or persistent/recurrent disease or in patients who did not respond to secondary BCG [14].

The treatment strategy of HGT1 is always challenged by the heterogeneous nature of the disease. This leads to a reasonable debate between bladder preservation and RC. The present study aimed to help determine the optimal treatment modality by comparing the outcomes, including the cancer-specific survival, of bladder preservation therapy with early or deferred RC.

## Materials and methods

Prospective data collection and follow-up were performed for patients undergoing TURBT for superficial UBC at a tertiary care center between January 2007 and January 2018. Histopathologically confirmed patients with HGT1 were included and divided into three groups according to the type of intervention, including conservative therapy and surveillance, early RC, and deferred RC.

Strategy of management

The management of HGT1 UBC in this tertiary care center followed international guidelines. A comprehensive history and thorough clinical examination were performed, followed by urethrocystoscopy and urine cytology for the initial evaluation of suspected bladder cancer. The macroscopic features of the tumor were recorded, including the tumor size, site, number, shape, and any associated mucosal abnormalities or carcinoma in situ (CIS). When indicated, a proper initial TUTBT was followed for the initial management and staging of the disease, and an en bloc resection of small papillary tumors with the underlying muscle layer and resection of larger tumors, in fractions, with the underlying muscularis propria and cauterization of the edges of the resection area was also performed. If the histopathology revealed HGT1 cancer, a repeated second-look TURBT was performed for more accurate staging, especially in cases with no detrusor muscle in the initial sampling. The presence of persistent tumors, the recurrence-free survival, and the response to BCG were all evaluated.

Surveillance plan

Follow-up cystoscopy and cytology were performed every three months for two years, then every six months until five years, and then annually thereafter. All patients with HGT1 were offered a six-week induction course of BCG (ImmuCyst® 81 mg of freeze-dried preparation made from the Connaught substrain of Bacillus Calmette-Guérin), followed by maintenance BCG, when appropriate. At each follow-up visit, a thorough clinical examination was performed to assess for disease recurrence and/or progression. Full lab work-ups were performed, including urinalyses, urine cultures and sensitivities, complete blood counts, renal and liver profiles, abdominal ultrasonography ± pelvic-abdominal computed tomography (CT), urethrocystoscopies, and urine cytologies. Plain chest X-rays or CT scans were performed when necessary. Following each session of BCG therapy, the symptoms and signs of any relevant adverse events were observed and recorded.

An early RC was performed directly after the diagnosis in patients with an incomplete TURBT due to an inaccessible endoscopic resection of the tumor or in those patients who had associated CIS. Patient characteristics and management strategies were collected, including age, sex, comorbid conditions, date of first diagnosis, dates and numbers of follow-up check cystoscopies ± TURBTs performed, induction and maintenance intravesical therapy, time to recurrence and/or progression, last follow-up visit, and survival status of the patients. In addition, the need for early versus deferred cystectomy was recorded, including indications, date of surgery, the interval from the last diagnosis, and follow-up data.

Deferred RC was defined as a cystectomy performed within six weeks after a failure of more than two consecutive six-week courses of intravesical therapy, including maintenance doses. The time between the cystectomy and the first TURBT and the number of TURBTs previously performed were recorded. Before RC, patients were thoroughly evaluated clinically, and all laboratory and imaging studies, including enhanced CT or magnetic resonance urography, were performed to further assess for metastasis. After the cystectomy, the patients were evaluated every three months during the first year and every six months thereafter.

Statistical analysis

Data analyses were performed using the commercially available Statistical Package for Social Science for Windows version 22 (SPSS, Armonk, NY: IBM Corp). Descriptive data were presented in terms of numbers and percentages or means ± SD or medians and ranges, depending on the data distribution. Fisher’s exact test, student’s t-test, and analysis of variance (ANOVA) or Mann-Whitney and Kruskal-Wallis tests were used to compare discrete and continuous variables, respectively. A Kaplan-Meier analysis was performed to assess cancer-specific survival, where any significant differences were assessed using the log-rank test. A two-sided p-value of less than 0.05 was accepted for statistically significant differences.

Ethics approval and consent to participate

The study was approved by the Committee of Bio-Medical Ethics of Umm Al-Qura University with approval number HAPO-02-K-012-2020-07-412. All participants gave written informed consent. All procedures performed in studies involving human participants were in accordance with the ethical standards of the institutional and/or national research committee and with the 1964 Helsinki Declaration and its later amendments.

## Results

Seventy-one patients with HGT1 UBC were included with a mean age of 53.6±12.5 years, including 88.7% males. The GI group included 34 (47.9%) patients who underwent conservative management, the GII group included 14 (19.7%) patients who underwent early RC, and the GIII group included 23 (32.4%) patients who underwent deferred RC. GII patients had a significantly higher number of CIS and multicentric tumors than the other two groups; otherwise, all the groups were comparable in their demographic and baseline criteria, including their age, sex, and tumor location (Table 1).

**Table 1:** Baseline demographic and tumor characteristics of both study groups *p<0.05 ** The total count is >100% due to the multiplicity in ≥2 locations CIS: carcinoma in situ; ERC: early radical cystectomy; DRC: deferred radical cystectomy; NA: not applicable

Variable		GI Conservative No (%)	GII ERC No (%)	GIII DRC No (%)	p-value
Total number of patients		34 (47.9)	14 (19.7)	23 (32.4)	NA
Mean age± SD		48± 16.2	53± 11.6	58± 16.4	0.06
Male gender		30 (88.2)	13 (92.8)	20 (87.0)	0.85
Tumor location**	Anterior wall	4 (11.8)	8 (57.1)	3 (13.0)	0.32
	Posterior wall	13 (38.2)	9 (64.3)	12 (8.7)	
	Right lateral wall	16 (47.1)	6 (42.8)	10 (4.3)	
	Left lateral wall	13 (38.2)	5 (35.7)	12 (4.3)	
	Domal	12 (5.9)	6 (42.8)	7 (30.4)	
Multicentric tumor		21 (61.8)	14 (100)	19 (82.6)	0.003*
Associated CIS		0 (0.0)	9 (64.3)	0 (0.0)	<0.001*

All of the patients in the GII group had multicentric tumors while 64.3% had CIS. The second-look cystoscopies revealed residual bladder tumors in 15 patients (21.1%), including five (35.7%) GII patients, six (17.6%) GI patients, and four (17.4%) GIII patients (p=0.64).

**Figure 1:**
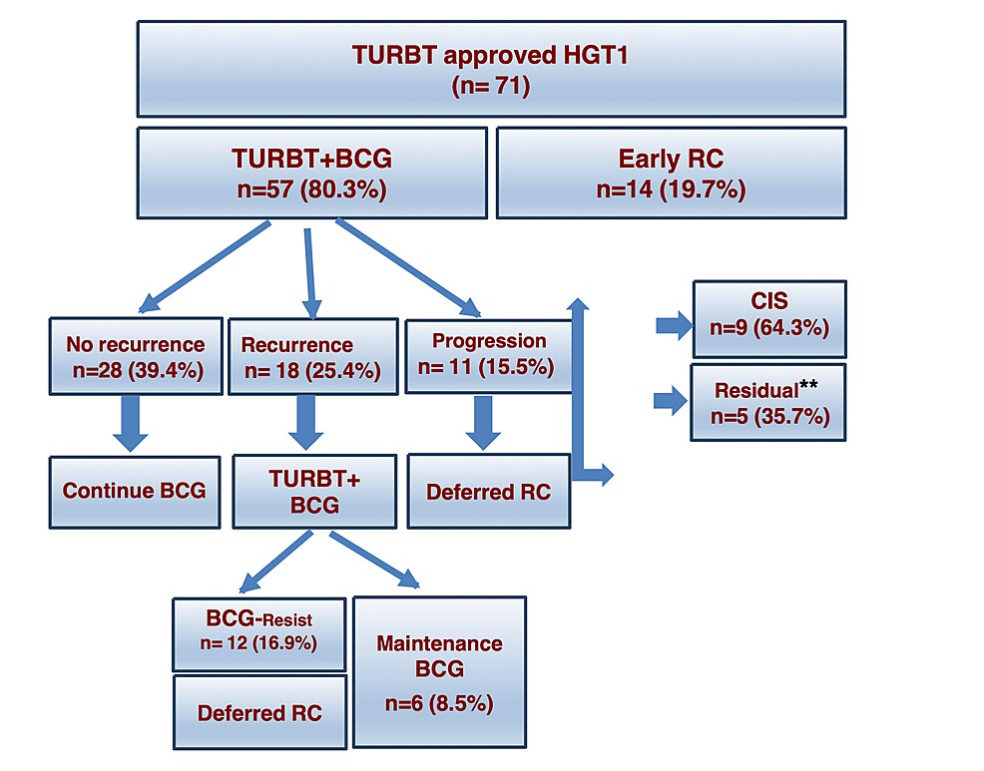
Flowsheet of the study population ** Residual tumors inaccessible for resection TURBT: transurethral resection of bladder tumor; RC: radical cystectomy; BCG: Bacillus Calmette Guerin

A flow chart of the management strategy of the study population showed that early RC was performed in 14 (19.7%) GI patients, including nine patients (64.3%) who had CIS and five patients (35.7%) with residual tumors, which were not accessible for endoscopic resection. Fifty-seven patients (80.3%) underwent TURBT followed by the induction of intravesical BCG therapy. Deferred RC was performed in 11 (15.5%) patients who had tumor progression to muscle-invasive disease and 12 (16.9%) patients who developed resistance to intravesical BCG immunotherapy (Figure 1). The remaining 34 (47.9%) patients continued with conservative treatment using maintenance BCG therapy according to international guidelines.

The median follow-up time was comparable between the patients undergoing early versus the patients having deferred RC (82 vs. 73 months, p=0.12). Compared to patients undergoing deferred RC, a significantly lower number of GII patients underwent more than two TURBTs (14.3% vs. 100%, p<0.001), and there was a significantly shorter time to cystectomy from the initial diagnosis (5 (3-7) vs. 36 (28-44) months, p=0.03). Ileal loop conduit and ileal orthotropic bladder diversion were performed in four (28.6%) vs. 11 (47.8%) and 10 (71.4%) vs. 12 (52.2%) patients (p=0.31) in the early and deferred RC groups, respectively. The number of postoperative adverse events and morbidity were comparable in the patients undergoing early versus the patients undergoing deferred RC (Table 2).

**Table 2:** Perioperative parameters of patients undergoing primary versus deferred cystectomy groups *Statistically significant difference; RC: radical cystectomy; TURBT: transurethral resection of bladder tumor; DVT: deep vein thrombosis; PE: pulmonary embolism

Variable		Early RC (n=14) No %	Deferred RC (n=23) No %	p-value
Median time of cystectomy from initial diagnosis/months (range)		5 (3-7)	36 (28-44)	0.03*
Median follow-up time/months (range)		82 (37-112)	73 (10-115)	0.12
No TURBTs	1-2	12 (85.7)	0 (0.0)	<0.001*
	>2	2 (14.3)	23 (100)	
Intraoperative adverse events		1 (7.1)	3 (13.0)	0.98
Urine diversion	Ileal-conduit	4 (28.6)	11 (47.8)	0.31
	Ileal orthotopic bladder	10 (71.4)	12 (52.2)	
Lymph node metastases	Positive	0 (0.0)	5 (21.7)	0.13
	Negative	14 (100)	18 (78.3)	
Postoperative morbidity	Wound sepsis	1 (7.1)	1 (4.3)	0.17
	Adhesive ileus	0 (0.0)	3 (13.0)	
	DVT/PE	0 (0.0)	2 (8.7)	
	Urinary leakage	0 (0.0)	3 (13.0)	
	Stricture uretero-ileal anastomosis	0 (0.0)	2 (8.7)	
Local recurrence (urethra or pelvis)		1 (7.1)	4 (17.4)	0.63
Distant metastasis		0 (0.0)	5 (21.7)	0.13
Adjuvant therapy		0 (0.0)	5 (21.7)	0.13

**Figure 2:**
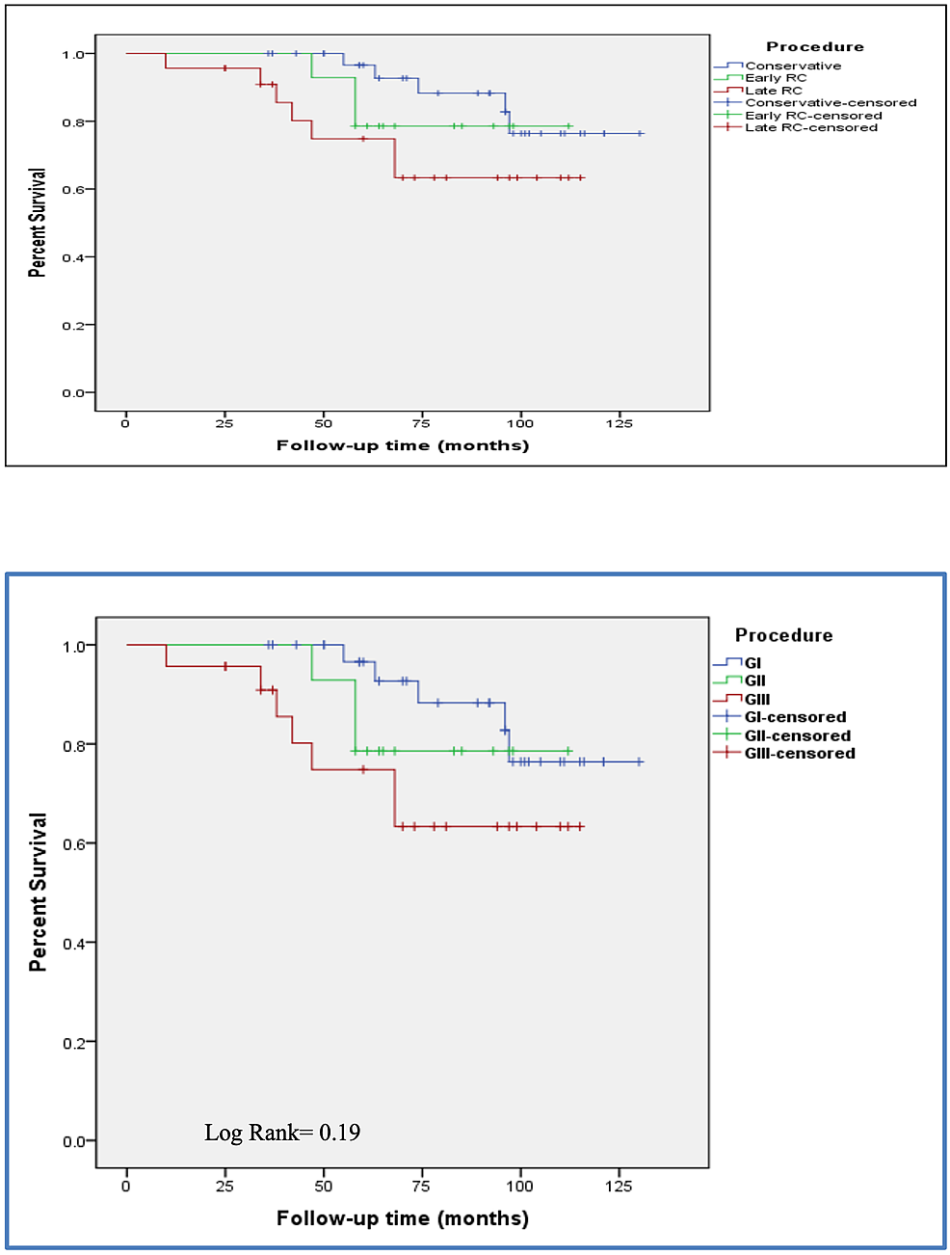
Cancer-specific survival for patients undergoing early versus deferred radical cystectomy

Although patients undergoing early RC had better CSS than those undergoing deferred RC, the difference did not reach statistical significance (log rank=0.19). The five-year and 10-year CSS±SE were 79%±3 vs. 75%±2 and 78%±4 vs. 64%±5, respectively (Figure 2). The five-year and accrual 10-year CSS±SE for patients with conservative treatment were 96%±2 and 76%±5, respectively (Figure 2).

## Discussion

For decades, intravesical BCG has been the gold standard treatment for HGT1 UBC. Despite the introduction of newer approaches for bladder preservation, RC cannot be ignored as an alternative treatment option due to the high risk of recurrence and progression, as well as disease-specific death, in these patients. Most guidelines recommended bladder preservation for a higher survival rate than RC and recommended a urinary diversion except for patients younger than 65 years old, where the overall survival after RC was comparable to conservative therapy. Therefore, deferred rather than immediate RC is warranted in patients with HGT1 who fail local therapy because bladder preservation therapy should be tried in these patients first [15]. Furthermore, RC is associated with psychological and surgical adverse events, and 80% of patients develop sexual dysfunction, which has a severe impact on quality of life [16-17]. Less than 5% of patients experience severe systemic life-threatening complications after intravesical BCG [18]. This would highlight the priority of conservative management for T1HG bladder cancer over RC.

In the present study, patients undergoing early RC had better five-year and 10-year CSS than those undergoing deferred RC. However, the difference did not reach statistical significance, which may be the result of the small sample size that could preclude any significant difference from appearing. However, the five-year CSS for patients with conservative treatment was better than that for those who underwent RC and comparable to the accrual 10-year CSS of those who underwent early RC. This is consistent with the guidelines available for the management of T1G3 noninvasive bladder carcinoma. Nevertheless, the results of CSS failed to confirm any significant difference between conservative management and RC except for the 15-year CSS, suggesting that RC may be a better management option for improving the long-term results [19].

In the current cohort, the increased rate of local recurrence and/or distant metastasis in 39% of patients undergoing deferred RC (despite not reaching statistical significance) may highlight the benefits of early RC. Only one patient (7%) in the early RC group who underwent ileal conduit diversion developed local urethral recurrence, and a urethrectomy was performed. Due to the high recurrence and progression rates, some authors recommended immediate cystectomy for patients with an excellent life expectancy and extensive or recurrent T1HG disease [20]. Similarly, other authors have observed that RC might be a better option for young patients and that bladder preservation can be kept as an option for elderly patients [21], especially because RC significantly improved the 10-year CSS in HGT1 patients. This may be explained by the associated CIS, which represents a known risk factor for high-grade tumors [22].

In a large series, early RC was associated with higher CSS at three years (84% vs. 79%), five years (78% vs. 71%), and 10 years (69% vs. 64%), although the differences were not statistically significant [23]. These figures were comparable to the results of the current cohort, despite the difference in sample size between both studies. In groups undergoing deferred RC in the latter study, survival was significantly lower in patients undergoing more than three TURBTs, and the authors recommended the adoption of conservative treatment for most of these patients [23].

In a recent meta-analysis of cohort studies, the CSS of bladder preservation was compared to that of RC. There were no significant differences between the treatment groups from the pooled odds ratio (OR) and 95% CI for two-year, five-year, and ten-year CSS, which were 0.78 (0.33-1.81, p= 0.56), 0.78 (0.44-1.38, p= 0.4), and 1.22 (0.59-2.52, p= 0.60), respectively [19]. Only one study favored the use of early RC compared to bladder preservation techniques more than three months after the diagnosis for improving the two-year and 10-year CSS, and this suggests that early RC could reduce the cancer-specific mortality compared to bladder preservation. Some authors showed significantly better five-year CSS in patients undergoing one or two TURBs (88% vs. 71%), in patients who had early RC within four months after the first TURB (86% vs. 77%), in patients who did not have adjuvant therapies (86% vs. 66%), in patients who had no tumor upstaging at the time of RC (89% vs. 67%), in patients who had bladder-confined tumors at the time of RC (88% vs. 56%) and in patients with no lymphadenopathy at the time of surgery (88% vs. 36%) [11]. Multivariate analysis showed that the number of TURBs (HR: 0.14), the time between the first TURB and RC (HR: 3.27), lymphadenopathy (HR, 0.13), and tumor stage at the time of RC (HR, 0.49) were independent predictors for the CSS [11]. Nevertheless, a pooled data analysis failed to find significant differences between conservative management and the RC approach in terms of the two-year, five-year, 10-year, and 15-year overall survival and progression-free survival, even when performing a limited subgroup analysis [19].

Both the survival and quality of life outcomes should be considered when choosing the appropriate treatment for HGT1 patients, depending on patient and tumor characteristics and life expectancy. All currently available guidelines recommend a thorough and close follow-up, regardless of the treatment option, for monitoring high-risk recurrence and progression for better survival outcomes [19]. In view of the continuous debate regarding choosing the appropriate treatment for HGT1 patients, a question is raised regarding the advantages of early RC in these patients. The literature is full of evidence that recommends RC in selected cases, and this is supported by the currently available guidelines.

RC provides the greatest hope for a cure, and the CSS is between 80-90%. Moreover, patients who are understaged have the best treatment response with aggressive management, especially the patients who are upstaged at the time of RC, which can range from 25-50% of the patients [24]. In addition, the associated lymphadenectomy improves the staging accuracy and may confer a therapeutic advantage. Some authors reported that 5-20% of these patients may conceal occult lymph node metastasis at the time of RC [25]. Furthermore, patients undergoing RC may avoid further intravesical therapy, which simplifies the follow-up regimen [26]. Nevertheless, only 4.7% of patients underwent RC within one year after the diagnosis of T1HG bladder cancer [27], despite the worse outcomes recently reported for patients having a deferred RC because most patients underwent RC after disease progression. These drawbacks of deferred RC include more upstaging, more lymph node-positive disease [28], and lower CSS rates, and more than one-third of these patients who were treated with deferred cystectomy die of metastatic disease [25].

The present cohort has limitations, including a relatively small sample size, which might preclude the validity of the statements regarding the statistical significance between the groups. However, as previously reported, using a randomized controlled methodology is unsuitable to evaluate this issue due to ethical considerations. Moreover, the ethnic background of the study population may affect the overall results when compared to worldwide studies. The patients’ and tumors’ characteristics might affect the heterogeneity of the reported data in different studies, where multifocal, high-grade large tumors may be associated with a higher malignant behavior. These parameters may also influence the response to specific therapies. The surgical and pathological skills of the team would strongly affect the development of early recurrence and the possibility of improper staging, and these can be influenced by an inadequate resection of the initial tumor, the missing of tumors or CIS, and an insufficient sampling of the muscularis propria. Nevertheless, this cohort further supports the previously discussed guidelines regarding the management of NMIBC.

## Conclusions

Early RC with urinary diversion should be considered an alternative treatment option in selected patients with HGT1, even after induction and maintenance of intravesical therapy, to avoid the significant risk of recurrence and progression. However, comparable CSS for early and deferred cystectomy in HGT1 bladder cancer would allow for priority in using the bladder preservation approach, especially in the elderly patients, and with close follow-up, it should be considered in all patients, regardless of the treatment options, for monitoring the patients with a high risk of recurrence and progression to help improve the survival outcomes. The inevitable limitations of the current small study necessitate the conduction of high-quality randomized controlled feasibility studies with an appropriate sample size. Till then, the management of these patients should be individualized.
